# A comparison of ultrasound measurements to assess carotid atherosclerosis development in subjects with and without type 2 diabetes

**DOI:** 10.1186/1476-7120-3-15

**Published:** 2005-06-15

**Authors:** Rebecca L Pollex, J David Spence, Andrew A House, Aaron Fenster, Anthony JG Hanley, Bernard Zinman, Stewart B Harris, Robert A Hegele

**Affiliations:** 1 Robarts Research Institute, London, Ontario, Canada; 2 Department of Medicine, University of Western Ontario, London, Ontario, Canada; 3 Department of Medicine, University of Toronto and Leadership Sinai Centre for Diabetes, Mount Sinai Hospital, Toronto, Ontario, Canada; 4 Thames Valley Family Practice Research Unit, University of Western Ontario, London, Ontario, Canada

## Abstract

**Background:**

Subjects with type 2 diabetes are at an increased risk of vascular complications. The use of carotid ultrasound remains an attractive, non-invasive method to monitor atherosclerotic disease progression and/or response to treatment in patients with type 2 diabetes, with intima-media thickness routinely used as the gold standard to detect pathology. However, alternative measurements, such as plaque area or volume, may represent a potentially more powerful approach. Thus, the objective of this study was to compare the traditional intima-media thickness measurement against the novel total plaque volume measurement in analyzing carotid atherosclerosis development in individuals with type 2 diabetes.

**Methods:**

The case-control study included 49 Oji-Cree adults with diabetes or impaired glucose tolerance, aged 21–69, and 49 sex- and age-matched normoglycemic subjects. At baseline, metabolic variables were measured, including body mass index, waist circumference, total cholesterol:high density lipoprotein ratio, plasma triglycerides, plasma glucose, and serum insulin. Carotid ultrasound measurements, 7 years later, assessed carotid arterial intima-media thickness and total plaque volume.

**Results:**

At baseline, the two groups were well matched for smoking habits, hypertension, body mass index, and waist circumference. Differences were noted in baseline measurements of total cholesterol:high density lipoprotein (*P *= 0.0006), plasma triglycerides (*P *< 0.0001) and fasting glucose (*P *< 0.0001). After seven years, carotid ultrasound scans revealed that total plaque volume measurements (*P *= 0.037), but not intima-media thickness measurements, were higher in subjects with diabetes/impaired glucose tolerance compared to the normoglycemic controls. Correlation between intima-media thickness and total plaque volume was moderate. Based on our study findings, to achieve power levels >0.70 when comparing intima-media thickness measurements for diabetics versus non-diabetics, thousands of study subjects are required. For comparing total plaque volume measurements, only hundreds of study subjects are required.

**Conclusion:**

The development of atherosclerotic plaque is greater in subjects with diabetes/impaired glucose tolerance. Total plaque volume appears to capture the atherosclerotic disease burden more effectively in subjects with type 2 diabetes, and would be an appropriate outcome measure for studies aimed at changing the diabetic milieu.

## Background

Macrovascular disease is the predominant contributor to morbidity and mortality in patients with type 2 diabetes [1]. Non-invasive assessment of arterial morphology using carotid ultrasound (US) represents an attractive tool to monitor progression and/or response to treatment in patients with type 2 diabetes. The traditional endpoint is intima-media thickness (IMT). Recently, however, newer measurements such as assessment of plaque area or volume represent a potentially more powerful approach, since this evaluates all plaques in the carotid system, and predicts clinical events somewhat more strongly than does IMT [2]. Thus, the objective of this case-control study was to compare the traditional IMT measurement versus the novel total plaque volume (TPV) measurement in analyzing carotid atherosclerosis development in individuals with type 2 diabetes.

## Methods

### Study Sample

Study participants were from the Oji-Cree community of Sandy Lake, Ontario, an isolated reserve located at the 55^th ^parallel of latitude, in the subarctic boreal forest of central Canada. Baseline demographic, clinical, and biochemical attributes were gathered during the Sandy Lake Health and Diabetes Project of 1993–1995, a prevalence study of type 2 diabetes [3]. Seven hundred and twenty eight members of this community (72% of the total population) aged 10 years and above, participated in the original survey. In a follow-up study initiated in 2001 [4], 278 adults free of coronary heart disease had US assessment of the carotid arteries. Of these, 161 had participated in the original prevalence study and had baseline measurements. For the current analysis, 49 subjects, 46 with type 2 diabetes and 3 with impaired glucose tolerance (IGT), were selected and matched for sex and age (± 3 years) with a normoglycemic control subject. Of the subjects with diabetes, 43.5% were receiving oral medication, and 4.4% were receiving insulin. For simplicity, from this point forward the 46 subjects with type 2 diabetes and 3 with IGT will be referred to as the "diabetic" group. Signed informed consent was obtained from all participants and study approval was granted by both the Sandy Lake First Nation Band Council and the institutional review boards of the University of Toronto and the University of Western Ontario.

### Clinical and biochemical baseline analysis

Body weight, height, waist circumference, and blood pressure were measured by standardized procedures [3]. Hypertensive individuals were defined as subjects having either a blood pressure reading >140/90 mmHg or taking anti-hypertensive medication. Measurements of fasting blood analytes, including triglycerides, insulin, lipoproteins, and total cholesterol were performed as described [3].

### Ultrasound examination

All subjects were examined using an HDI 5000 scanner equipped with Sono-CT compound imaging and a L12-5 transducer (Advanced Technology Laboratories, Bothell, Washington) that had been flown to the community and housed within the Diabetes Research Center. Common carotid US images for all participants were gathered over a 4-week period and from this data, IMT, total plaque area (TPA) and TPV measurements were determined. TPA was strongly correlated with TPV in these subjects (r = 0.921, *P *< 0.0001), and thus for simplicity, only TPV measurements were compared against IMT.

### IMT measurement

IMT was determined as previously described [5,6]. Briefly, a single observer, blinded to subjects' vascular risk, measured combined thickness of intima and media of the far wall of both common carotid arteries. Images were recorded from an anterolateral longitudinal view. The still images were analyzed using computerized edge-detection software (Prowin™) [7]. Using a step-wise algorithm, conditional sets of "edges" (consisting of lumen-intima and media-adventitia echoes) were located within the image and then tested for "edge strength", with the subsequent deletion of weak edge points. Once all acceptable edge points were identified, boundary gaps were filled by linear interpolation. The distance between lumen-intima and media-adventitia boundaries was then measured to calculate IMT. Mean IMT was computed from 120 measurements over a 10 mm span ending 5 mm proximal to the transition between the common carotid and bulb regions. Intra- and inter-operator coefficients of variation of 3.0 and 3.1%, respectively and intra- and inter-operator intraclass correlations were both 0.97 [n = 50] (both P < 0.01).

### TPV measurement

TPV was determined as previously described [5,6]. Briefly, 3D ultrasound images were acquired with a mechanical linear scanning system and analyzed with L3Di visualization software [Life Imaging Systems Inc., London, Ontario]. Plaque volumes were measured using manual planimetry: each 3D image was 'sliced' transversely at an inter-slice distance of 1 mm, moving from one plaque edge to the other. Plaque boundaries were traced using a mouse driven cross-haired cursor. Slice areas were summed and multiplied by inter-slice distance to calculate plaque volume. For this analysis, TPV was defined as the sum of all plaque volumes on one side between the clavicle and angle of the jaw. Intra- and inter-observer reliability were 0.94 [n = 40] and 0.93 [n = 40], respectively (both P < 0.01).

### Statistical analysis

SAS version 8.2 (SAS Institute, Cary, NC) was used for all statistical comparisons. Data are presented as means ± SE. The distribution of BMI, plasma total cholesterol, triglycerides, high density lipoprotein (HDL), and serum insulin, were non-normal in this data set, and thus were logarithmically transformed (natural log) and subjected to analysis of normality. IMT and TPV were also normalized using the inverse transformation of IMT and the square root transformation of TPV. The transformed variables were used for parametric statistical analyses, but the untransformed values are presented in Table 1. For continuous variables, differences between the groups were tested by the Student's *t *test; categorical variables were tested by *γ*^2 ^analysis. Statistical significance was taken at nominal *P *< 0.05 for all comparisons. Correlation analysis between IMT and TPV was performed using Pearson correlation analysis.

**Table 1:** Clinical and biochemical attributes of Oji-Cree at baseline and carotid measurements after 7 years

	Diabetic subjects	Non-diabetic subjects	P-value
number/females	49/26	49/26	
			
attributes at screening			
age (years)	40.3 ± 1.8	40.4 ± 1.8	NS (0.96)
duration of diabetes (years)	2.20 ± 0.62	-	-
current smokers (%)	18.4	10.2	NS (0.25)
hypertensive (%)	36.7	32.7	NS (0.67)
antihypertensive treatment (%)	16.3	8.2	NS (0.22)
body mass index (kg/m^2^)	29.6 ± 0.5	29.5 ± 0.6	NS (0.75)
waist circumference (cm)	101 ± 1.4	99.8 ± 1.5	NS (0.63)
TC:HDL ratio	4.95 ± 0.18	4.10 ± 0.17	0.0006
plasma triglycerides (mmol/L)	2.25 ± 0.14	1.53 ± 0.11	<0.0001
plasma glucose (mmol/L)	10.79 ± 0.63	5.53 ± 0.07	<0.0001
serum insulin (pmol/L)	157 ± 12	133 ± 9	NS (0.16)
			
time elapsed since screening (years)	7.34 ± 0.10	7.33 ± 0.10	NS (0.96)
mean IMT (*μ*m)	795 ± 19	789 ± 26	NS (0.49)
mean TPV (mm^3^)	109.9 ± 23.0	64.0 ± 17.0	0.037

Data are means ± SE unless otherwise indicated.

Abbreviations: TC, total cholesterol; HDL, high density lipoprotein; IMT, intima-media thickness; TPV, total plaque volume; NS, not significant

Hypothetical sample sizes were calculated using the online calculator for normal power calculations (normal distribution 2-sample equal variances) found at the UCLA Department of Statistics website [8]. This statistical tool calculates the sample size for two-sided tests of hypotheses on normal means, when the common population standard deviation is known, using the following formula:



where n_1 _and n_2 _are the sample sizes of the two groups, u_*α*/2 _and u_*β *_are the lower limits of the cumulative standard normal probability integrals, *σ *is the known common standard deviation, *δ*_0 _is the least favourable non-negative difference consistent with the test hypothesis, and *δ*_1 _is difference in the population means [9].

Using the normalized transformed means from the case-control study, the mean standard deviation as the common standard deviation (SD), and a significance level of 0.05, sample sizes were calculated. Transformed means and standard deviations were 1.29 (diabetic) *vs *1.33 (non-diabetic), SD 0.237, for IMT (inverse transformation), and 7.81 (diabetic) *vs *4.94 (non-diabetic), SD 6.71, for TPV (square root transformation). Power was tested at 0.70, 0.80 and 0.90.

## Results

### Clinical and biochemical attributes of Oji-Cree subjects at baseline

As presented in Table 1, at the initial time of screening, the average age of the study participants was 40.3 ± 1.8 years for subjects with diabetes, and 40.4 ± 1.8 years for the control subjects. 53.1% were females. Subjects with diabetes were relatively newly diagnosed, with an average diabetes duration of 2.20 ± 0.62 years. Comparing the baseline characteristics of the diabetic and non-diabetic subjects, there was no significant difference in terms of smoking, hypertension, body mass index (BMI), waist circumference, or serum insulin concentrations. However, both TC:HDL ratio and plasma triglyceride and glucose concentrations were elevated in the diabetic subjects (*P *= 0.0006, *P *< 0.0001, and *P *< 0.0001, respectively).

### Carotid plaque measurements after 7 years

**Figure 1:**
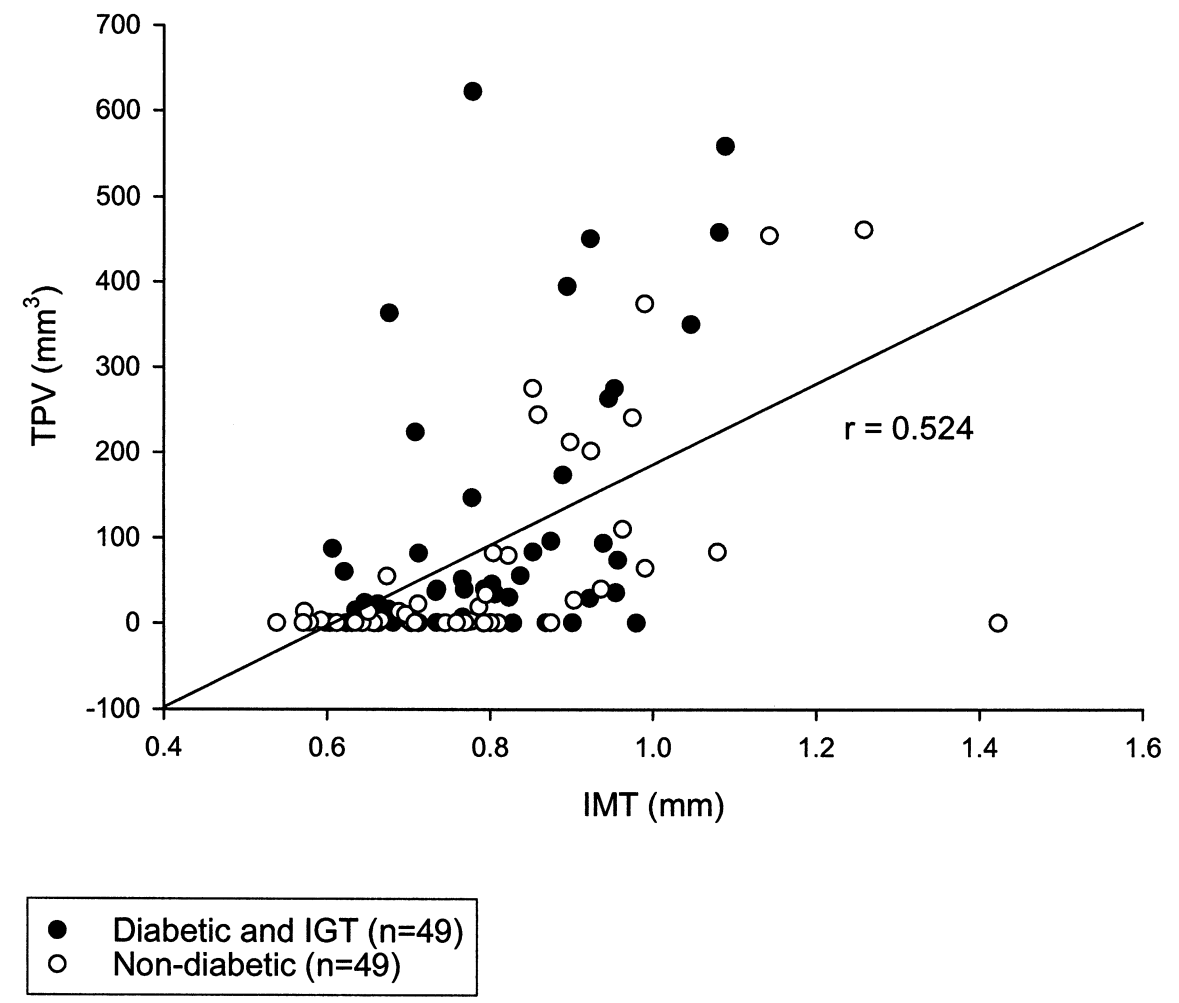
**Correlation between carotid arterial total plaque volume (TPV) and intima-media thickness (IMT). **Subjects with type 2 diabetes or IGT are represented with black dots (n = 49), while non-diabetic control subjects are represented with white dots (n = 49). The Pearson correlation coefficient (r) = 0.524.

Following 7 years, mean IMT values were slightly elevated for subjects with diabetes (795 ± 19 *μ*m) *vs *non-diabetic subjects (789 ± 26 *μ*m), however the difference was not significant (*P *= 0.49). Mean TPV measurements, however, were significantly higher in subjects with diabetes (109.9 ± 23.0 mm^3 ^*vs *64.0 ± 17.0 mm^3^, *P *= 0.037). The simple Pearson correlation coefficient between untransformed IMT and TPV was 0.524 (*P *< 0.0001) (Figure 1); the simple Pearson correlation coefficient between transformed values (1/IMT and square root of TPV) was approximately the same (r = -0.556, *P *< 0.0001).

### Comparison of sample sizes required for hypothetical studies

Using the mean IMT and TPV results from our case-control study as primary endpoints, we determined the sample sizes that would be required for a hypothetical study to achieve a statistical power of 0.70, 0.80 and 0.90. As shown in Table 2, to detect a 6 *μ*m difference in IMT, a total of 1202 subjects would be required for a 0.70 power level, 1528 subjects for a 0.80 power level, and 2044 subjects for a 0.90 power level. To detect a 45.9 mm^3 ^difference in TPV, a total of 136 subjects would be required for a 0.70 power level, 172 subjects for a 0.80 power level, and 230 subjects for a 0.90 power level.

**Table 2:** Comparison of sample sizes required for hypothetical trials with mean IMT or TPV as primary endpoints

Endpoint	Significance Level	Power	N_diabetic_	N_non-diabetic_	N _total_
					
IMT (inverse): 6 *μ*m difference					
	0.05	0.70	601	601	1202
	0.05	0.80	764	764	1528
	0.05	0.90	1022	1022	2044
					
TPV (square root): 45.9 mm^3 ^difference					
	0.05	0.70	68	68	136
	0.05	0.80	86	86	172
	0.05	0.90	115	115	230

Abbreviations: IMT, intima-media thickness; TPV, total plaque volume

## Discussion

We report: 1) elevated TPV for diabetic subjects *vs *non-diabetic subjects following a 7 year period (*P *= 0.037); 2) increased sensitivity of the TPV measurement in comparison to IMT measurements for diabetic subjects.

A previous study by Hunt *et al*., convincingly showed that early atherogenesis is present before the onset of diabetes, and thus is not solely dependent on the clinical manifestation of diabetes [10], but rather, both conditions (diabetes and cardiovascular disease) originate from a "common soil" of pro-inflammatory and pro-atherogenic risk factors [11]. Our study examined atherosclerosis burden after the diagnosis of diabetes had been made and found that diabetic subjects had TPV measurements that were 1.7-fold higher than non-diabetic subjects (*P *= 0.037). Similar observations have been made previously, such as in the Insulin Resistance Atherosclerosis Study (n = ~1200), where subjects with diabetes had increased carotid wall thickness at baseline (~70 *μ*m increase in common carotid, ~130 *μ*m increase in internal carotid) [12], and, over a five year time period, had IMT progression rates approximately twice as high as non-diabetic subjects (7.2 ± 1.9 *vs *3.8 ± 1.3 *μ*m/year) [13]. The Bruneck Study (n = 826) found that type 2 diabetes was a strong independent predictor (OR = 5.0, *P *< 0.001) of US-determined, advanced stenotic atherosclerosis, defined by >40% lumenal narrowing [14].

Significant differences were also noted in the lipid profile of diabetic subjects *vs *controls, with a greater TC:HDL ratio and elevated triglycerides observed for those with diabetes. Glucose intolerance has been previously reported as an independent predictor of both triglycerides and HDL cholesterol [15]. This worsening of lipids with glucose intolerance may potentially explain the differences between the two groups in terms of plaque volume progression.

While a significant difference was found for TPV, no significant difference was found for IMT between diabetic and non-diabetic subjects, although IMT tended to be greater for diabetic subjects. The lack of a significant difference undoubtedly is related to the low number of subjects, but it is apparent that it may also be due to the relative insensitivity of carotid IMT as a surrogate marker for atherosclerosis in patients with type 2 diabetes. The potential increased sensitivity for TPV was reflected by our finding of a significant difference for a relatively small study sample. An important feature for enhanced sensitivity is found in the wider dynamic scale ranges for TPV compared to IMT: ~90% of the IMT measurements fall within a relatively narrow 0.55–1.0 mm range, whereas ~60% of TPV values fall within a range of 5–500 mm^3^. Thus, the dynamic range of measurements varied by ~100-fold for TPV compared to ~2-fold for the IMT. Furthermore, the quantity being measured (mm^3 ^for TPV *vs *mm for IMT) is much larger for TPV, so that in relation to the resolution of the ultrasound method, TPV is much easier to quantify both accurately and reliably.

In designing studies it may be worthwhile to consider using TPV in addition to the traditional IMT measurement, as a primary endpoint, due to its potentially greater sensitivity and discrimination, which may have the benefit of greater statistical power, allowing for the use of a small sample size. For example, to observe a significant difference in IMT using values similar to those observed in this case-control study, (ie. 6 *μ*m difference in mean IMT) (Table 2), thousands of subjects are required to achieve a statistical power of even 0.70. In contrast, only a few hundred subjects are required to observe a statistical power of 0.90 for the TPV difference seen in our study (45.9 mm^3^). Performing studies with over a thousand subjects [10,13] imposes limitations and difficulties, which could be minimized by using a more sensitive technique such as TPV measurement. Studies on the effects of therapy on atherosclerosis using TPV as the outcome have already been effectively carried out with much smaller sample sizes. Ainsworth *et al. *[16] have shown significant differences between active atorvastatin and placebo in 3 months, with a sample size of only 20 per group.

IMT and TPV, however, are not interchangeable. The correlation between IMT and TPV in this study, although statistically significant, was moderate (r<0.7) and, as has been noted previously, these different US-derived measures of carotid artery morphology likely represent distinct attributes of atherosclerosis [5]. IMT may reflect wall hyperplasia or hypertrophy related to hypertension [17] and TPV may reflect the later stages of plaque formation and the total carotid disease burden in a subject [5]. This may be more relevant for the disease process of diabetes. It is also important to note that in our previous work [5] IMT correlated better with hypertension and age, and as blood pressure and age were balanced between the groups, this might explain the lack of difference observed here for IMT. It may be the case that IMT would capture the atherosclerotic disease burden more effectively in hypertensive diabetics, and would be a more appropriate outcome measure for studies aimed at improving hypertension in diabetics. These implications must be taken into consideration when designing and analysing a study, and more research is needed to provide a complete understanding of how these US measures fit into the pathophysiology of atherosclerotic disease.

Considering that by nature atherosclerotic plaque is not evenly distributed along the arterial wall, it is logical to develop methods that will attempt to quantify the total plaque burden more accurately. With the relatively larger volumes being measured for TPV assessment *vs *IMT or plaque area measurements, there are the accompanying benefits of more statistical power and less patients required per study, and also the potential benefit of less time required to observe significant differences between study groups. However, at present, using TPV as an assessment tool remains a labour-intensive task and has the additional disadvantage of not yet being widely used and standardized. Furthermore, in studies among children or other very young subjects, ultrasound evaluation of the arteries may be limited to IMT simply because plaque would not yet be developed in most cases. However, some plaque can be identified in most subjects above age 35 or 40 [2].

## Conclusion

In conclusion, this case-control study showed significantly greater carotid TPV measurements in subjects with type 2 diabetes *vs *subjects without type 2 diabetes, after a 7-year period. This supports previous reports of increased carotid ultrasound analytes for persons with type 2 diabetes, but additionally, this study highlights the effectiveness of TPV as a marker for atherosclerotic disease burden in diabetes, and encourages the further use and development of this robust measurement. Currently the determination of TPV requires labour-intensive manual tracing, which has proven to be an accurate and reliable measurement [18,19]. Semi-automated methods are now in development. However, for TPV to be used as a universally implemented research/clinical tool, further studies will be needed to clarify the relationships among IMT, TPV and perhaps coronary plaque volume measured by intravascular ultrasound.

## Competing interests

The author(s) declare that they have no competing interests.

## Authors' contributions

RLP participated in the design of the study, analysis of the data, and writing of the manuscript. JDS, AAH, AF, AJGH, BZ, and SBH provided patients and data for the study, and assisted with manuscript revisions. RAH participated in the design of the study and writing of the manuscript. All authors read and approved the final manuscript.

## References

[B1] (2005). Centers for Disease Control and Prevention, US Department of Health and Human Services. Diabetes: Diabling, Deadly, and on the Rise.

[B2] Spence JD (2002). Ultrasound measurement of carotid plaque as a surrogate outcome for coronary artery disease. Am J Cardiol.

[B3] Hanley AJG, Harris SB, Barnie A, Gittelsohn J, Wolever TMS, Logan A, Zinman B (1995). The Sandy Lake Health and Diabetes Project: design, methods and lessons learned. Chronic Dis Canada.

[B4] Hanley AJG, Harris SB, Mamakeesick M, Goodwin K, Fiddler E, Hegele RA, McLaughlin JR, Zinman B (2003). Complications of type 2 diabetes among Native Canadians: increasing our understanding of prevalence and risk factors. Canadian J Diabetes.

[B5] Al-Shali K, House AA, Hanley AJ, Khan HM, Harris SB, Mamakeesick M, Zinman B, Fenster A, Spence JD, Hegele RA (2005). Differences between carotid wall morphological phenotypes measured by ultrasound in one, two and three dimensions. Atherosclerosis.

[B6] Hegele RA, Al-Shali K, Khan HM, Hanley AJG, Harris SB, Mamakeesick M, Zinman B, Fenster A, Spence JD, House AA (2005). Carotid ultrasound in one, two and three dimension. Vasc Dis Prevention.

[B7] Selzer RH, Hodis HN, Kwong-Fu H, Mack WJ, Lee PL, Liu CR, Liu CH (1994). Evaluation of computerized edge tracking for quantifying intima-media thickness of the common carotid artery from B-mode ultrasound images. Atherosclerosis.

[B8] UCLA Department of Statistics. http://calculators.stat.ucla.edu/powercalc/normal/n-2-equal/.

[B9] Mace AE (1974). Sample-Size Determination.

[B10] Hunt KJ, Williams K, Rivera D, O'Leary DH, Haffner SM, Stern MP, Gonzalez Villalpando C (2003). Elevated carotid artery intima-media thickness levels in individuals who subsequently develop type 2 diabetes. Arterioscler Thromb Vasc Biol.

[B11] Stern MP (1995). Diabetes and cardiovascular disease. The "common soil" hypothesis. Diabetes.

[B12] Wagenknecht LE, D'Agostino RBJ, Haffner SM, Savage PJ, Rewers M (1998). Impaired glucose tolerance, type 2 diabetes, and carotid wall thickness: the Insulin Resistance Atherosclerosis Study. Diabetes Care.

[B13] Wagenknecht LE, Zaccaro D, Espeland MA, Karter AJ, O'Leary DH, Haffner SM (2003). Diabetes and progression of carotid atherosclerosis: the insulin resistance atherosclerosis study. Arterioscler Thromb Vasc Biol.

[B14] Bonora E, Kiechl S, Oberhollenzer F, Egger G, Bonadonna RC, Muggeo M, Willeit J (2000). Impaired glucose tolerance, Type II diabetes mellitus and carotid atherosclerosis: prospective results from the Bruneck Study. Diabetologia.

[B15] Harris SB, Zinman B, Hanley A, Gittelsohn J, Hegele R, Connelly PW, Shah B, Hux JE (2002). The impact of diabetes on cardiovascular risk factors and outcomes in a native Canadian population. Diabetes Res Clin Pract.

[B16] Ainsworth CD, Blake CC, Tamayo A, Fenster A, Spence JD (2005). Measurement of change in carotid plaque volume: A 3-dimensional ultrasound tool for rapid evaluation of new therapies. Stroke.

[B17] Fujii K, Abe I, Ohya Y, Ohta Y, Arima H, Akasaki T, Yoshinari M, Iida M (2003). Risk factors for the progression of early carotid atherosclerosis in a male working population. Hypertens Res.

[B18] Fenster A, Landry A, Downey DB, Hegele RA, Spence JD (2004). 3D ultrasound imaging of the carotid arteries. Curr Drug Targets Cardiovasc Haematol Disord.

[B19] Landry A, Spence JD, Fenster A (2004). Measurement of carotid plaque volume by 3-dimensional ultrasound. Stroke.

